# Assessment of myocardium at risk with contrast enhanced steady-state free precession cine cardiovascular magnetic resonance compared to single-photon emission computed tomography

**DOI:** 10.1186/1532-429X-12-25

**Published:** 2010-04-30

**Authors:** Peder Sörensson, Einar Heiberg, Nawsad Saleh, Frederic Bouvier, Kenneth Caidahl, Per Tornvall, Lars Rydén, John Pernow, Håkan Arheden

**Affiliations:** 1 Department of Medicine, Karolinska Institutet, Karolinska University Hospital, Stockholm, Sweden; 2 Molecular Medicine and Surgery, Karolinska Institutet, Karolinska University Hospital, Stockholm, Sweden; 3 Lund University Hospital, Department of Clinical Physiology, Lund, Sweden

## Abstract

**Background:**

Final infarct size following coronary occlusion is determined by the duration of ischemia, the size of myocardium at risk (MaR) and reperfusion injury. The reference method for determining MaR, single-photon emission computed tomography (SPECT) before reperfusion, is impractical in an acute setting. The aim of the present study was to evaluate whether MaR can be determined from the contrast enhanced myocardium using steady-state free precession (SSFP) cine cardiovascular magnetic resonance (CMR) performed one week after the acute event in ST-elevation myocardial infarction (STEMI) patients with total coronary occlusion.

**Results:**

Sixteen patients with STEMI (age 64 ± 8 years) received intravenous 99 m-Tc immediately before primary percutaneous coronary intervention. SPECT was performed within four hours. MaR was defined as the non-perfused myocardial volume derived with SPECT. CMR was performed 7.8 ± 1.2 days after the myocardial infarction using a protocol in which the contrast agent was administered before acquisition of short-axis SSFP cines. MaR was evaluated as the contrast enhanced myocardial volume in the cines by two blinded observers. MaR determined from the enhanced region on cine CMR correlated significantly with that derived with SPECT (r^2 ^= 0.78, p < 0.001). The difference in MaR determined by CMR and SPECT was 0.5 ± 5.1% (mean ± SD). The interobserver variability of contrast enhanced cine SSFP measurements was 1.6 ± 3.7% (mean ± SD) of the left ventricle wall volume.

**Conclusions:**

Contrast enhanced SSFP cine CMR performed one week after acute infarction accurately depicts MaR prior to reperfusion in STEMI patients with total occlusion undergoing primary PCI. This suggests that a single CMR examination might be performed for determination of MaR and infarct size.

## Background

The extent of myocardial injury following myocardial infarction (MI) is an important determinant of short and long term prognosis [1]. Early reperfusion, either by pharmacological thrombolysis or by primary percutaneous coronary intervention, is therefore mandatory for the effective myocardial salvage [2,3]. The introduction of reperfusion therapy has lead to considerable improvement in the survival of patients with acute ST-elevation MI (STEMI) [4]. Still many patients develop extensive myocardial damage underlining the need for therapeutic modalities that limit the extent of the final injury. The development of such treatment is dependent on methods that accurately determines the size of the jeopardized myocardium at risk (MaR) and that are feasible to use in patients presenting with STEMI.

MaR comprises both myocardial tissue that is irreversibly injured at the time of reperfusion and a viable border zone of reversibly injured cells [5]. The difference between MaR and infarct size is used to calculate the myocardial salvage index which is a measurement of the effectiveness of interventions that aim to reduce the extent of the final myocardial infarct [6]. The reference method for determining MaR is single photon emission computer tomography (SPECT) [7-9]. This method requires injection of a labelled isotope before reperfusion which, besides being difficult to accomplish in an acute setting, may delay the time to the coronary intervention and exposes the patient to additional radiation. These factors clearly limit the use of SPECT in clinical trials and illustrate the need for development of new methods to determine MaR.

Cardiovascular magnetic resonance (CMR) has an excellent in-plane spatial resolution, producing precise measurement of infarct size [10,11] without exposing the patient to ionizing radiation. In 2005 Laissy et al. evaluated the diagnostic value of contrast enhanced (CE) time-resolved balanced steady-state free precession (SSFP) in the assessment of infarct size compared with late gadolinium enhancement (LGE) sequences [12]. They found a close correlation and concluded that "CE cine-SSFP sequences should play a role in assessing necrotic and jeopardized myocardium after acute MI". Experimental [13-15] and clinical [16-19] studies suggest that MaR can be detected with different T2-weighted CMR depicting the initial oedema several days after the acute MI. T2-weighted imaging for quantification of MaR was recently validated in humans using myocardial perfusion SPECT [20]. Alternatively, it has been suggested that unenhanced T1-weighted images can be used to quantify oedema [21], infarcted endocardial surface area (infarct-ESA) [22] or single-shot dark blood-prepared SSFP [23] for potential subsequent identification of MaR.

We used a modified CMR protocol in which gadolinium contrast agent is injected just before SSFP-imaging is commenced for LV volumes and function, which is an important distinction from standardized methods [24]. Using this protocol we observed transmural contrast enhancement seemingly representing the MaR on cine-SSFP sequences. This CMR protocol would thereby have the potential to determine MaR and infarct size in one single examination performed several days after the acute event if the observed transmural contrast enhancement on SSFP images represents MaR.

This study therefore tested the hypothesis that the observed contrast enhancement on cine SSFP one week after an acute MI represents MaR by comparing it with reference method myocardial perfusion SPECT obtained before opening the coronary occlusion.

## Materials and methods

### Study Protocol

Sixteen consecutive patients, age 64 ± 8, with first time STEMI admitted for primary percutaneous coronary intervention (PCI) during the period February 2007 to December 2008 when myocardial perfusion scintigraphy was accessible and who fulfilled the inclusion and exclusion criteria were enrolled. Twelve patients were included at the Karolinska University Hospital and four at the Lund University Hospital. Myocardial perfusion isotope was mostly available during daytime, which limited the inclusion rate. Data from the four patients from Lund constitute part of an earlier published study [20]. Inclusion criteria were: chest pain ≥ 30 minutes and ≤ 9 hours duration, ST-elevation in at least two contiguous ECG leads or left bundle branch block and a complete coronary occlusion (TIMI flow grade 0) of the infarct-related artery at the time of coronary angiography. Exclusion criteria were prior history of MI, prior coronary artery by-pass grafting, cardiogenic shock, known renal insufficiency, contraindications for CMR, permanent atrial fibrillation. The study was approved by the independent local ethics committee at each center. Written informed consent was obtained from all patients. The protocol was designed, conducted and analysed with Good Clinical Practice regulation.

### Coronary Angiography

Coronary angiography was performed to confirm complete coronary occlusion in the infarct related artery and coronary angioplasty was then performed according to local standard procedures at the discretion of the individual physician. The intervention was completed by a coronary angiogram to determine final TIMI grade flow.

### SPECT

Prior to opening of the occluded vessel the patients received a body weight-adjusted (350-700 MBq) iv injection of 99 mTc tetrofosmin (Amersham Health, Buckinghamshire, UK) or sestamibi (MIBI, Cardio-lite, Bristol Myers Squibb, USA). Myocardial perfusion SPECT imaging was performed within four hours to visualize and quantify MaR using either of two dual-head cameras: GE camera (Ventri, GE Healthcare) or Sopha camera (DST-XL; Sopha Medical Vision, Bue Cedex, France). The patients were placed in the supine position and imaged in steps of 5.6 degrees using a 64 × 64 matrix, with a typical pixel size of 5 × 5 mm and a slice thickness of 5 mm. The reconstructed voxel size was 3 × 3 × 3 mm (Sopha) or 6.4 × 6.4 × 6.4 mm (GE). Image acquisition time was approximately 15 min. Iterative reconstruction using maximum-likelihood expectation maximization was performed with a low-resolution Butterworth filter and a cut off frequency set to 0.5 of Nyquist and an order of 5.0. No attenuation or scatter correction was applied and short-axis images were reconstructed semi-automatically on the respective workstation for each camera.

### CMR

A standard clinical CMR protocol, except for the time of administration of contrast, was scheduled to be performed one week after the onset of symptoms. Timing was chosen at one week to avoid the early infarct phase where a rapid decrease in infarct size has been reported [25]. Two 1.5 T systems were used: Signa Excite TwinSpeed (General Electric Healthcare, Waukesha, WI, USA) or Philips Intera CV (Philips, Best, Nederlands). Eight- (GE) and five-channel (Philips) cardiac-coil was used and all patients were in the supine position with vector-ECG monitoring. A bolus of gadolinium contrast agent (0.2 mmol/kg bodyweight (Omniscan, GE Healthcare, Norway or Magnevist, Bayer Pharma, Berlin, Germany) was given iv just before positioning the patient in the scanner. The image protocol included scout images, localization of the short axis and then covering the whole left ventricle (LV) with retrospectively gated SSFP cines. The following typical parameters on GE-scanner was used; SSFP (TE 1.58 ms, TR 3.61 ms, flip angle 60 degrees, 25 phases, 8 mm slice, no gap, matrix 226 × 226). LGE images were acquired 15-20 minutes after contrast injection using an inversion recovery gradient echo sequence (TE 3.3 ms, TR 7.0 ms, TI 180-250 ms to null the myocardium, 8 mm slice, no gap, matrix 256 × 192) and the same slice orientation as cine SSFP images. Typical parameters on the Philips scanner was; SSFP (TE 1.4 ms, TR 2.8 ms, flip angle 60 degrees, 30 phases, 8 mm slice, matrix 160 × 141). LGE images were acquired 15-20 minutes after contrast injection using inversion recovery gradient echo sequence (TE 1.14 ms, TR, 3.8 ms, TI 180-250 ms, 8 mm slice, no gap, matrix 240 × 180). Cardiac triggering was set for diastole to reduce motion artefacts. Each slice was obtained during end-expiratory breath holding. Two-, three- and four chamber views were also obtained to confirm the findings.

### Image analysis and evaluation

Analysis of myocardial perfusion SPECT defect for MaR was performed off-line using freely available segmentation software (Segment v1.702; http://segment.heiberg.se)[26,27]. The automatic segmentation finds the centerline through the left ventricular wall and identifies the endo- and epicardium based on an individually estimated wall thickness and signal intensity values within the image [28]. Manual adjustment of the automatic delineation was sometimes required in the left ventricular outflow region. The perfusion defect was determined by an automated algorithm that considers myocardium with <55% of normal counts as being ischemic [29]. MaR was quantified as % of the left ventricle.

CMR images were analysed off-line using the same software Segment. End-diastolic and end-systolic volumes, ejection fraction, stroke volume and left ventricular volume was calculated on SSFP cines. The contrast enhanced myocardial volume in SSFP cines was manually delineated in end-diastole and end- systole by two observers blinded to each other's findings and to SPECT results. The values were averaged for internal control and expressed as % of the LV wall volume. See additional file 1: Movie1 for the original data used to perform this analysis. The relatively low contrast difference between contrast in injured myocardium and remote myocardium precluded the use of a semi-quantification algorithm. Infarction was quantified, in a later session, using an automated quantification method [30] that has been validated in ex vivo and in vivo experiment in which partial volume effects are accounted for.

To determine the image relative intensity ratio between enhanced and remote myocardium a region of interest (ROI) was manually drawn in approximately 80% of the enhanced area in three consecutive slices and the same size ROI was drawn in the remote myocardium.

### Cardiac enzymes

Cardiac biomarkers were sampled every fourth hour after admission on day one and every sixth hour on day two. Troponin-T was analysed with immunoassay technique (Modular Analytics E-module, Roche Diagnostics) and CK-MB was analysed using chemical luminescence technique (UniCel DxI 800, Beckman Coulter AB).

### Statistics

All data are expressed as mean and standard deviation. Wilcoxon Mann-Whitney Rank Sum test was used to test for differences between groups (SPECT and contrast enhanced SSFP). Wilcoxon Sign Rank test was used to test the relative signal enhancement in contrast enhanced regions on SSFP images compared to remote myocardium. Bland-Altman plots were constructed for comparing contrast enhanced cine SSFP images with reference standard myocardial perfusion SPECT. Interobserver variability was calculated as the standard deviation of the difference between two blinded observers divided by the average of the two observers. A two-sided P-value < 0.05 was considered statistically significant. Statistical analysis was performed using commercially available software GraphPad Prism version 5.00 for Windows (GraphPad Software, San Diego, CA, USA, http://www.graphpad.com). The authors had full access to the data and take responsibility for its integrity. All authors have read and agreed to the manuscript as written.

## Results

### Patients

The culprit lesion was located in the right coronary artery (RCA) in twelve patients, in the left anterior descending artery (LAD) in two and in the left circumflex artery (LCx) in one (Table 1). All patients received oral antiplatelet therapy with loading doses of 320-500 mg aspirin and 300 or 600 mg clopidogrel before the intervention and iv infusion of a GPIIb/IIIa inhibitor in connection with the PCI procedure. TIMI flow grade 3 was achieved in the affected artery in all patients. The time from onset of chest pain to reperfusion was 222 ± 120 minutes. The maximum levels of troponin-T and CK-MB were 7.0 ± 5.6 μg/l and 252 ± 210 μg/l, respectively. There were no severe adverse events or reinfarctions prior to the CMR investigation that was performed 7.8 ± 1.2 days after the onset of symptoms.

**Table 1:** Myocardium at risk comparing SPECT and CMR. Infarct size measured with LGE and myocardial salvage index

	Culprit lesion	MaR SPECT (LV%)	MaR CMR (LV%)	Infarct size LGE (LV%)	Salvage Index (%)
Patient 1	RCA	38	31	15	52
Patient 2	LAD	51	47	30	36
Patient 3	RCA	30	35	1	97
Patient 4	RCA	30	30	9	70
Patient 5	RCA	31	28	14	50
Patient 6	RCA	15	23	2	91
Patient 7	LAD	37	32	11	66
Patient 8	RCA	26	24	9	63
Patient 9	RCA	25	21	9	57
Patient 10	RCA	28	29	7	76
Patient 11	RCA	11	17	10	41
Patient 12	RCA	26	24	10	58
Patient 13	RCA	14	23	5	78
Patient 14	RCA	18	24	9	63
Patient 15	CX	28	27	9	67
Patient 16	RCA	22	27	1	96

RCA = right coronary artery; LAD = left ascending artery; CX = left circumflex artery;

MaR = Myocardium at Risk; SPECT = single-photon emission computed tomography;

CMR = cardiovascular magnetic resonance; LV = left ventricle. Myocardial salvage index

(1-scar/MaR).

### CMR & SPECT

**Figure 1:**
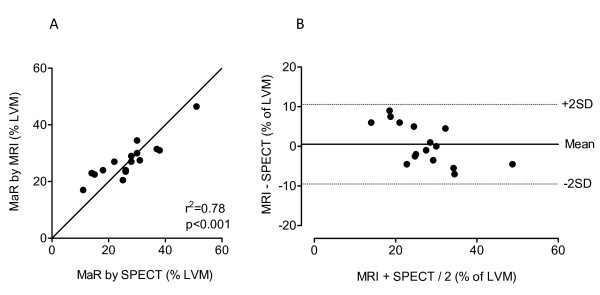
**Agreement between MaR determined by CMR and SPECT**. Panel A: Scatter plot showing MaR one week after reperfusion determined by gadolinum enhanced SSFP cines plotted versus MaR as it was before reperfusion determined by myocardial perfusion SPECT together with best line and line of identity. Panel B: Bland-Altman plot showing the agreement between MaR determined by myocardial perfusion SPECT and contrast enhanced cine SSFP. The difference was 0.5 ± 10% (mean ± 2SD).

**Figure 2:**
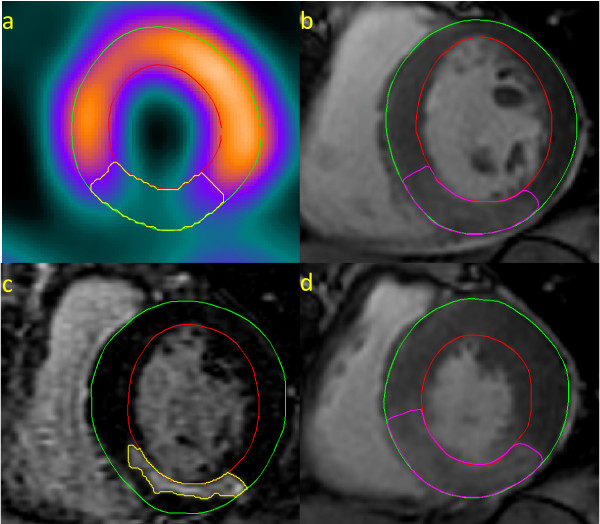
**Inferior STEMI**. Corresponding left ventricular short axis views from a patient with inferior STEMI. MaR determined by (a) myocardial perfusion SPECT, (b) gadolinium enhanced SSFP at end-diastole, (c) infarct size images with LGE and (d) gadolinium enhanced SSFP at end-systole.

**Figure 3:**
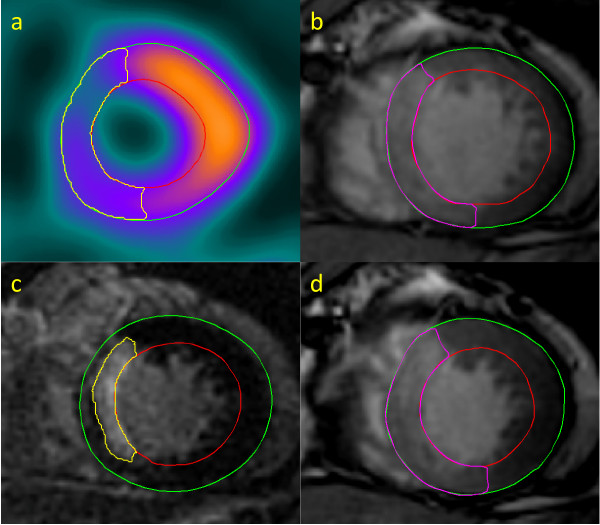
**Anterior STEMI**. Corresponding left ventricular short axis views from a patient with anterior myocardial STEMI. MaR determined by (a) myocardial perfusion SPECT, (b) gadolinium enhanced SSFP at end-diastole, (c) infarct size images with LGE and (d) gadolinium enhanced SSFP at end-systole. It is clearly seen that the region of gadolinium enhancement does not correspond in size or endocardial extent to the region of myocardium at risk either by SSFP cines or myocardial SPECT.

Images of good diagnostic quality were obtained in all patients. Throughout the entire RR-interval a high transmural signal was consistently observed in the infarct region, and the infarct was always within the area of contrast enhancement in the SSFP cines. MaR, defined as the non-perfused myocardial volume on SPECT, ranged from 11 to 51% (mean 27 ± 10%) of the LV wall volume. The contrast enhanced region on SSFP cines, calculated as the mean values obtained at end-diastole and end-systole, ranged from 17 to 47% (mean 27 ± 7%) of the LV wall volume. As illustrated in Figure 1A there was a good correlation between MaR determined from the enhanced region on SSFP cines and that determined with SPECT (r^2 ^= 0.78, p < 0.001). The difference between the enhanced region on SSFP cines and MaR on SPECT was 0.5 ± 5.1% (p = 0.60, Figure 1B). The location of the enhanced region on SSFP cines always agreed with MaR on myocardial perfusion SPECT images. Two typical examples of MaR and infarct area in the RCA and LAD regions are shown in Figures 2 and 3. The signal intensity ratio between regions of gadolinium enhanced and remote myocardium was 1.42 ± 0.25 (p < 0.001). The interobserver variability for gadolinium enhanced myocardium between two readers was 1.6 ± 3.7%. Infarct size determined by CMR ranged from 1 to 30% (mean 9 ± 7%) of LV wall volume and mean transmurality ranged from 26 to 52%.

## Discussion

This study demonstrates that the contrast enhanced myocardium on SSFP cines represents the MaR as determined by myocardial perfusion SPECT. Thus gadolinium enhanced CMR performed one week after an acute STEMI can accurately determine MaR simultaneously with the infarct size.

**Figure 4:**
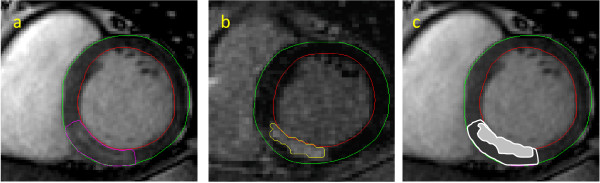
**Myocardial salvage**. Corresponding left ventricular short axis views from a patient with inferior STEMI. MaR determined by (a) gadolinium enhanced SSFP at end-diastole, (b) infarct size images with LGE and (c). The infarcted area from panel b is superimposed on MaR from panel a (light grey shade on dark striped area). Myocardial salvage index is calculated as (1-scar/MaR).

The extent of the final infarct size is dependent on several factors including the duration of ischemia, the degree of collateral flow, myocardial oxygen demand and the size of MaR [1]. In studies aimed at limiting infarct size, accurate determination of MaR is crucial in order to calculate the myocardial salvage index (Figure 4). The reference standard method for determination of MaR is SPECT which has the important limitation that the isotope needs to be prepared and injected before the ischemic myocardium is reperfused. Since isotope for SPECT may not be readily available in the acute setting of primary PCI, new methods which are more feasible to use in patients with acute MI need to be developed.

Our results differ from those of an earlier study in 2005 where there was a good correlation between gadolinium SSFP cines and infarct size on LGE [12]. These differences may in part be attributable to differences in study populations with less transmural enhancement on gadolinium SSFP cines in the present study likely due to shorter pain-to-opening times Another possible explanation may be that our study included only STEMI patients.

Previous attempts to investigate MaR through CMR focused on different T2-weighted sequences. It was suggested that MaR can be estimated using T2-weighted imaging with short inversion time inversion-recovery (STIR) or T2-prepared single-shot SSFP or a combination of both [13,17,18,22]. The mechanism is not fully known but oedema caused by the transient ischemia, cellular swelling and impaired microvascular reperfusion have been suggested [31]. T2-weighted imaging to quantify MaR was first validated in humans using myocardial perfusion SPECT as reference [20]. The ACUT_2_E TSE-SSFP study recently showed promising results by using a hybrid method of T2-weighting with bright-blood contrast in dogs [15].

The presently described technique also has the advantage of sequentially determining MaR and the final infarct size with excellent spatial resolution in one single examination. Since SSFP cines are available on all CMR-scanners, MaR assessed by gadolinium SSFP cines might be used as a robust independent complement in cases where T2-weighted imaging may be difficult or subjected to artefact problems. Fewer acquisitions would be required since the volumetric dataset for MaR would be acquired during the normal cine acquisitions for cardiac mass and function.

The mechanism behind the enhanced myocardium observed using gadolinium SSFP cines and the gadolinium kinetics is not clarified by the present investigation and needs to be investigated in further studies. The balanced cine SSFP sequence is known to generate T2/T1-weighting. Bright signal is due to short T1- or long T2-relaxation or a combination of the two. Gadolinium based contrast agents such as Gd-DTPA increases the relaxation rates by approximately the same amount. On a percentage basis, Gd-DTPA alters the T1-relaxation to a much larger extent than T2-relaxation, since T1 in tissue is much longer than T2 [32]. With an increased concentration of contrast medium in the MaR, an increase in signal would be seen from early balanced cine SSFP echoes (i.e. proton spins that have not experienced T2-relaxation), while signal originating from subsequent echoes (i.e. proton spins that have experienced T2-relaxation) would remain approximately constant. As seen in the studies using T2-weighted sequences to estimate MaR, the bright signal seen in MaR is largely due to the prolonged T2-relaxation of the oedema. This is most likely the case using balanced cine SSFP as well, but the signal is "boosted" by the shortening of T1-relaxation in the MaR.

The present results are of potential clinical and scientific importance because it provides an easily accessible technique for quantification of the efficacy of reperfusion therapy by calculation of the myocardial salvage index [33]. The estimation of MaR and final infarct size might be achieved in a stable patient situation several days after the acute MI. This is in contrast to SPECT which requires preparation and injection of isotope before reperfusion and image acquisition within a few hours in an unstable patient.

### Limitations

The low number of patients may be seen as a limitation to this study. On the other hand the material includes a large span of myocardium at risk ranging from 11-51% of the LV wall volume, which is important for the evaluation. We did not use a semi-quantitative method to determine the contrast enhanced myocardium on SSFP cines due to the relatively low signal increase compared to remote myocardium. No comparison was made to T2-weighted sequences because of low number of patients and that it was not the objective with this study. Other limitations are that only four out of sixteen patients were female, most infarctions involved the RCA territory and only total coronary occlusions (TIMI flow grade 0) were included.

## Conclusions

Contrast enhanced SSFP cines performed one week after the acute event accurately depicts MaR as it was before reperfusion in STEMI patients with total occlusion undergoing primary PCI. A single CMR examination can thereby be performed for determination of MaR and infarct size.

## Competing interests

The authors declare that they have no competing interests.

## Authors' contributions

PS enrolled the majority of the patients. PS, EH and HA participated in the design of the study and coordination, performed the statistical analysis and drafted the manuscript. NS, FB, KC, PT, LR, JP participated in the design of the study and coordination and helped to draft the manuscript. All authors read and approved the final manuscript.

## Supplementary Material

Additional file 1 **An example of gadolinium enhanced SSFP cine of myocardium at risk in an inferior infarction one week after admission. **The inferior midventricular wall enhancement represents MaR which can be seen both in end-diastole and end-systole. Normal myocardial signal in anterior, septal and lateral walls. Click here for file
